# Apnea-Hypopnea Index in Chronic Obstructive Pulmonary Disease Exacerbation Requiring Noninvasive Mechanical Ventilation with Average Volume-Assured Pressure Support

**DOI:** 10.1155/2021/7793657

**Published:** 2021-11-27

**Authors:** Killen H. Briones-Claudett, Adela Romero Lopez, Mónica H. Briones-Claudett, Mariuxi del Pilar Cabrera Baños, Killen H. Briones Zamora, Diana C. Briones Marquez, Andrea P. Icaza-Freire, Luc J. I. Zimmermann, Antonio W. D. Gavilanes, Michelle Grunauer

**Affiliations:** ^1^Universidad de Guayaquil, Facultad de Ciencias Médicas, Guayaquil, Ecuador; ^2^Physiology and Respiratory-Center Briones-Claudett, Guayaquil, Ecuador; ^3^Intensive Care Unit, Ecuadorian Institute of Social Security (IESS), Babahoyo, Ecuador; ^4^Universidad Espíritu Santo, Samborondón, Ecuador; ^5^Pneumology Department, Ecuadorian Institute of Social Security (IESS), Guayaquil, Ecuador; ^6^Intensive Care Unit, Santa Maria Clinic, Guayaquil, Ecuador; ^7^School of Medicine, Universidad San Francisco de Quito, Quito, Ecuador; ^8^School for Oncology & Developmental Biology (GROW), University of Maastricht, Maastricht, Netherlands; ^9^Critical Care Department, Hospital de Los Valles, Quito, Ecuador

## Abstract

**Introduction:**

This study intends to determine the Apnea-Hypopnea Index in patients hospitalized with acute hypercapnic respiratory failure from chronic obstructive pulmonary disease exacerbation, who require noninvasive ventilation with average volume-assured pressure support (AVAPS), as well as describes the clinical characteristics of these patients.

**Materials and Methods:**

We designed a single-center prospective study. The coexistence of Apnea-Hypopnea Index and clinical, gasometric, spirometric, respiratory polygraphy, and ventilatory characteristics were determined. The clinical characteristics found were categorized and compared according to the Apnea-Hypopnea Index (AHI) < 5, AHI 5–15, and AHI >15. A *p* value <0.05 was considered statistically significant.

**Results:**

During the study period, a total of 100 patients were admitted to the ICU with a diagnosis of acute hypercapnic respiratory failure due to COPD exacerbation. 72 patients presented with acute respiratory failure and fulfilled criteria for ventilatory support. Within them, 24 received invasive mechanical ventilation and 48 NIV. After applying the inclusion criteria for this study, 30 patients were eligible. An AHI >5 was present in 24 of the 30 patients recruited (80%). Neck circumference (cm), Epworth scale, and Mallampati score evidenced significant differences when compared to the patient's AHI <5, AHI 5–15, and AHI >15 (*p* < 0.05). Furthermore, patients with an AHI >5 had longer hospital admissions, prolonged periods on mechanical ventilation, and a higher percentage of intubation rates.

**Conclusion:**

Apnea-Hypopnea Index and chronic obstructive pulmonary disease exacerbation are a frequent association found in patients with acute hypercapnic respiratory failure and COPD exacerbations that require NIV. This association could be a determining factor in the response to NIV, especially when AVAPS is used as a ventilatory strategy.

## 1. Introduction

The main clinical condition for the use of NIV in patients with acute hypercapnic respiratory failure due to exacerbated chronic obstructive pulmonary disease (COPD) is alveolar hypoventilation, which originates from an imbalance between the respiratory muscles capacity to maintain ventilation and gas exchange, resulting in hypercapnia [1].

Sleep disturbance is one of the most common symptoms reported in these patients, occurring in 40% of them in one large study [2]. Moreover, the apnea-hypopnea syndrome (AHS) is a condition characterized by increased upper airway resistance associated with an intermittent decrease or absence of inspiratory airflow, often causing arousals during sleep. Furthermore, the increase in upper airway resistance, classically seen in obstructive sleep apnea (OSA), impacts the diaphragmatic effort and the ability to maintain adequate ventilation during sleep [3].

The combination of COPD and apnea-hypopnea syndrome has been named “overlap syndrome” [4]. However, the term “overlap syndrome” could also apply to the coexistence of sleep apnea and any chronic respiratory disease, but it is used to define the association between OSA and COPD exclusively. Sleep apnea can be divided according to the Apnea-Hypopnea Index (AHI) in no detectable events or up to AHI <5 events/hour; mild: IAH 5 to 15 events/hour; moderate: AHI >15 to 30 events/hour; and severe: AHI >30 events/hour [5].

Studies that investigate sleep apnea in patients with COPD or COPD in patients with sleep apnea have been performed for patients in stable conditions [6, 7]. Despite this, studies that report sleep apnea in patients with COPD exacerbations are very limited [8, 9]. To our knowledge, there is no current study that identifies patients in the intensive care unit under noninvasive mechanical ventilation **(**NIV) with COPD and AHI.

Average volume-assured pressure support (AVAPS) as a ventilatory strategy has been demonstrated to be useful in patients with chronic respiratory insufficiency [10, 11]. In addition, Briones Claudett et al. [12] reported benefits in a patient with COPD and hypercapnic encephalopathy. Furthermore, Cao et al. [13] described the results of the use of AVAPS in 58 subjects with acute hypercapnic respiratory failure (AHRF) and COPD, while Çiftci et al. [14] showed that average volume-assured pressure support and bilevel positive airway pressure (BiPAP S/T-AVAPS) were effective and well-tolerated in 76.4% of cases of patients with COPD.

In this context, this study was designed to determine the presence and clinical evolution of OSA in patients hospitalized with acute hypercapnic respiratory failure due to COPD exacerbation receiving NIV with AVAPS in the intensive care unit (ICU). The primary endpoint was as follows: what is the percentage of the Apnea-Hypopnea Index in patients hospitalized with COPD exacerbation receiving NIV with AVAPS in the ICU? The secondary endpoints were as follows: describing the clinical characteristics of the AHI in these patients and the days of hospital stay including NIV and intubation rates.

## 2. Materials and Methods

This is a single-center prospective study. All the patients were admitted from July 1, 2013, to February 28, 2014. Informed consent was obtained from patients and their subrogates if they were not able to respond by themselves.

Written consent was obtained for all patients to participate.

The study was approved by the ethics committee of the Santa Maria Hospital, approved number: N/REFE: 18-2-2013; protocol/serial/number 2013 (2) [15].

A total of 30 patients with a diagnosis of acute hypercapnic respiratory failure due to COPD exacerbation who required NIV with average volume-assured pressure support (AVAPS) were recruited for this study.

### 2.1. Inclusion and Exclusion Criteria

The inclusion criteria were as follows: (a) age: 18 and older; (b) admitted to the intensive care unit of Santa Maria Clinic; (c) patients with ventilatory failure secondary to hypercapnia with carbon dioxide concentration (PaCO_2_) > 45 mmHg, the negative logarithm of the hydrogen ion concentration (pH) 7.35 or less; (d) patients with inadequate oxygenation, partial pressure of arterial oxygen (PaO_2_) < 60 mmHg breathing ambient air, and arterial oxygen saturation (SaO_2_) < 92%; (e) severe dyspnea, respiratory rate (RR) > 25 breaths per minute, and use of accessory muscles during hospitalization.

Contrastingly, patients were excluded if they had hemodynamic instability and were noncooperative or agitated, unable to use the interface device, had recent surgery of the upper airway tract, or used NIV with a do-not-resuscitate (DNR) order, as well as patients who had received some type of sedation during the study period or if a respiratory polygraphy could not be done.

### 2.2. The Protocol of Noninvasive Mechanical Ventilation for the Treatment of Acute Respiratory Failure

When a patient was diagnosed with acute respiratory failure (ARF) in the emergency department, a senior physician was consulted for assessment and management and decided about admission to the ICU and initiation of NIV and adjusted the ventilatory parameters. Patients were observed and evaluated by respiratory therapists, resident doctors, and nurses trained in NIV. NIV with average volume-assured pressure support (AVAPS) was used.

Patients were placed in spontaneous/timed modes with AVAPS with a maximum programmed inspiratory positive pressure (IPAP) of 20 cm H_2_O and a minimum programmed IPAP of 12 cm H_2_O and positive expiratory pressure (EPAP) of 6 to 8 cm H_2_O. The programmed tidal volume was 8 mL/kg of the predicted body weight calculated from measured height (kg) using the following formula: male: PBW = 50 + 0.91 × (height in cm–152.4) and female: PBW = 45.5 + 0.91 × (height in cm–152.4).

Other parameters were respiratory rate of 12–14 breaths/min; the rise time was 300 to 400 ms; the inspiratory time was 0.8 to 1.2 s. Additionally, O_2_ supplements were added through an O_2_ adapter close to the mask to keep the SaO_2_ above 90%. The maximum IPAP, exhaled tidal volume (EVT), minute volume (*V*_min_), and leakages were controlled through the ventilator software. The BiPAP synchrony with AVAPS and Autotrak (Respironics Inc., Murrysville, Pennsylvania, USA) was used along with a series of Mirage IV (ResMed) face masks.

In addition to ventilatory support, both groups received bronchodilators, intravenous corticosteroids, and antibiotic therapy consisting of a beta-lactam in combination with a fluoroquinolone.

### 2.3. Measurements

ABG was measured at the baseline of NIV use. Additionally, any complications related to the interphase (mask) were documented, and the mask use and tolerability related to excessive discomfort, nasal ulcer, gastric distension, and claustrophobia were evaluated as well. The following parameters were also collected: tidal volume (TV), IPAP, VTE, *V*_min_, leakage, RR, and positive end-inspiratory pressure.

The measured data were as follows: age, sex, and arterial blood gas measurements at the beginning of the NIV protocol (pH, pCo_2_, concentration of buffer or blood bicarbonate **(**HCO_3_), and base excess), SaO_2_, HR, RR, systolic blood pressure (SBP), diastolic blood pressure (DBP), and radiological alterations including intercurrent disease (described as alterations in 1, 2, 3, and 4 quadrants), body mass index, and neck circumference. The Epworth sleepiness scale and Mallampati score [16] are described below and are applied in this study.

The arterial blood gas (ABGs) measurements were taken before and during treatment with NIV; the ventilatory parameters used were mode (spontaneous, spontaneous/time, AVAPS), positive inspiratory pressure (IPAP), and positive expiratory pressure (EPAP). Further, the type of mask used was Mirage Series IV (ResMed). All the patients were evaluated by respiratory therapists under the strict supervision of trained NIV physicians.

### 2.4. Discontinuance of Therapy with NIV

The process of weaning off the NIV was initiated when clinical stability was achieved, which was defined as RR less than 24 breaths/min and improvement of SaO_2_ > 92%, with a percentage of inspired FiO_2_ below 35%. Once the patient remained stable, NIV was withdrawn.

### 2.5. NIV Withdrawal

Clinical stability was defined as (1) RR < 25 rpm; (2) heart rate (HR) < 90 bpm; (3) compensated arterial pH with SaO_2_ (%) > 90% in ambient air or with low flow oxygen (<3 L per minute).

### 2.6. Follow-Up and Measurements during Hospitalization

Pre- and postbronchodilator spirometry, using 400 mcg of salbutamol inhaled by MDI along with nightly sleep apnea monitoring, was obtained from patients before hospital discharge.

We used a spirometer with a turbine transducer DATOSPIR 70 (SILBELMET W-10) SIBEL S.A., Barcelona, Spain, and polygraphy equipment (ApneaLink, ResMed) for these measurements.

### 2.7. Definitions and Diagnostic Criteria

Sleep apnea syndrome was defined according to the 2012 American Academy of Sleep Medicine [17].

Apnea is defined as the absence or reduction in the amplitude of the respiratory flow signal over 90%, measured using an oronasal thermal sensor, lasting more than 10 seconds. Furthermore, apnea is obstructive if accompanied by a respiratory effort measured in the thoracoabdominal bands; it is central in the absence of such respiratory effort and is mixed if it starts as central and ends with respiratory effort [18].

Hypopnea is a reduction of the respiratory flow signal equal to or greater than 30% and less than 90% of more than 10 seconds, which is accompanied by an arterial oxygen desaturation equal to or greater than 3% in a microawakening on the electroencephalogram (EEG) or both [19].

### 2.8. The Apnea-Hypopnea Index (AHI)

The AHI is defined as the sum of the total number of apnea and hypopnea episodes per hour of sleep calculated during the total sleep time.

### 2.9. Severity of IAH

The polygraph test was performed in the stable phase before the patient was discharged from the hospital and measured by respiratory polygraphy.

The severity of the AHS is based on the number of episodes of apnea and hypopnea per hour of sleep [20, 21]. It is classified as normal: no detectable events or up to AHI <5 events/hour; mild: IAH 5 to 15 events/hour; moderate: AHI >15 to 30 events/hour; or severe: AHI >30 events/hour.

### 2.10. COPD Definition and Severity

The airflow limitation was measured by spirometry [22].

### 2.11. ApneaLink

The ApneaLink device is a single-channel screening tool for sleep apnea consisting of a nasal cannula attached to a small case that houses a pressure transducer. The device is held in place by a belt worn around the user's chest [23, 24]. The ApneaLink device operates on battery power and has a sampling rate of 100 Hz with a 16 bit signal processor. The internal memory storage is 15 MB, which allows for approximately 10 hours of data collection. The ApneaLink software analyzes data generated by the ﬂow signal, producing a 1-page report. Full disclosure of data is available for review and rescoring by the clinician. The ApneaLink does not discriminate obstructive from central events because the signal is based only on airﬂow and there is no recording of respiratory effort.

Additionally, the ResMed ApneaLink software system version 9.0. Tm Plus-Germany ResMed. was used. The measurements through the ApneaLink were made before hospital discharge and with patients breathing room air.

### 2.12. Outcome Measures

#### 2.12.1. Primary Endpoint

What is the percentage of the AHI in patients hospitalized with COPD exacerbation receiving NIV with AVAPS in the ICU?

#### 2.12.2. Secondary Endpoints

It is to describe the clinical characteristics of AHI in these patients and days of hospital stay, including NIV and intubation rates.

### 2.13. Statistical Analysis

All data were expressed as means ± standard deviation (SD) for continuous variables and as percentages for categorical variables. The test for independent samples was used on the data with a Gaussian distribution and similar variance (determined through homogeneity of variance or the Levene test). A nonparametric test (chi-square or Fisher's exact test) was used on the data with a nonnormal distribution for categorical variables. A *p* value of <0.05 was considered statistically significant.

## 3. Results

**Figure 1:**
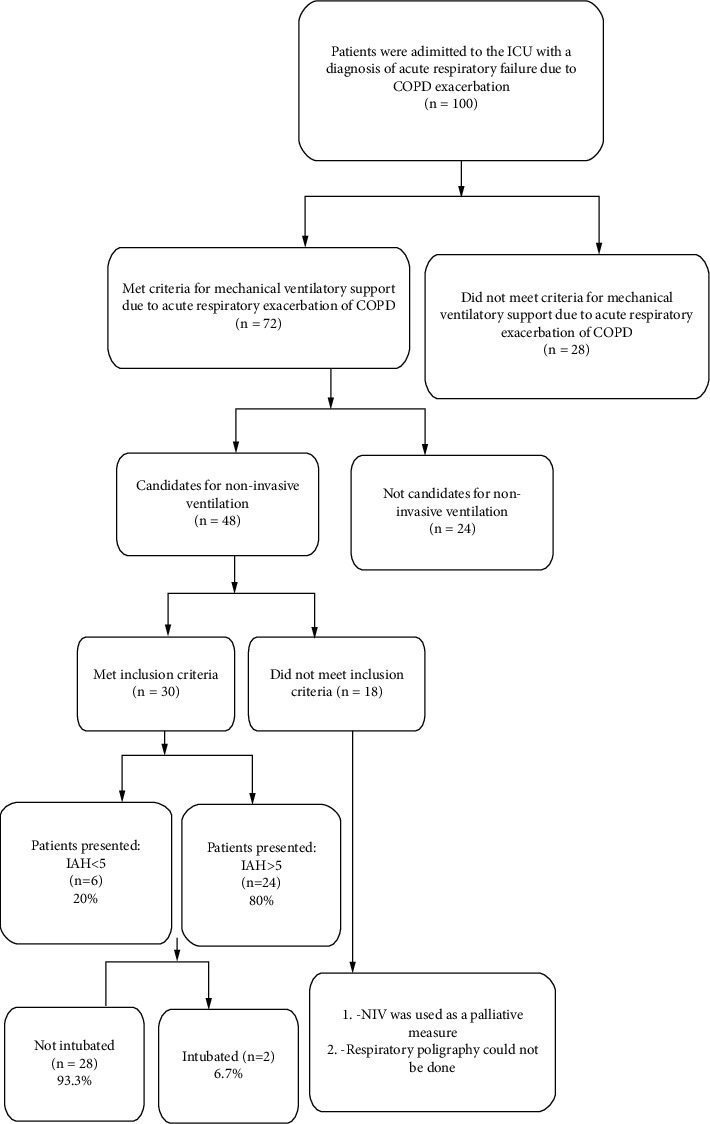
Flow chart of selection and presentation of patients.

During the eight months of study period, a total of 100 patients were admitted to the ICU with diagnosis of acute respiratory failure due to COPD exacerbation. Of these, 72 had a diagnosis of severe acute respiratory failure, and 24 patients received conventional treatment that included IMV while 48 patients were candidates for NIV. 30 patients met the inclusion/exclusion criteria of this study and were followed up with spirometry and respiratory polygraphy testing. See Figure 1.

The most frequent age of presentation was 72.6 ± 14.3 SD. Body mass index was 25.4 ± 5.2 SD. The baseline characteristics of the population are described in Table 1.

Patients were divided according to the severity of the AHI: < 5 (*n* = 6), of 5–15 (*n* = 13), and >15 (*n* = 11).

Neck circumference (cm), Epworth scale, and Mallampati score presented significant differences when compared to the patient's AHI <5, AHI 5–15, and AHI >15 (*p* < 0,05). See Table 2. Furthermore, patients with an AHI >5 had longer hospital admissions, prolonged periods on mechanical ventilation, and a higher percentage of intubation rates. See Table 3.

## 4. Discussion

We found a high presence of AHI in patients with acute respiratory hypercapnic failure due to COPD exacerbation who required NIV with a high percentage of success of NIV with the ventilatory strategy of AVAPS. On the other hand, AHI >5 was present in 24 of 30 patients (80%).

Studies that investigate AHI in patients with COPD have been performed in stable clinical conditions. However, the coexistence of OSA in the general population is variable and some studies report a high prevalence in patients with moderate to severe COPD [25, 26].

During sleep, several physiological variations occur including a decrease in ventilation (15–20%), reduced metabolic rate (10–15%), increased airway resistance, increased pCO_2_ (2 to 8 mmHg), decreased PaO_2_ (3 to 10 mmHg), and decreased SaO_2_ (±2 mmHg) [27]. Furthermore, the presence of OSA in patients with COPD increases the risk of exacerbation and shortens the time to the first exacerbation [28].

Our study identifies patients with AHI + COPD exacerbation with acute hypercapnic respiratory failure requiring NIV in a population that has not been previously studied. Few studies have evaluated the preexistence of undetected OSA in hospitalized patients, but some reports result in obese patients [29] or patients with decompensated heart failure [30].

In our study, we found a high percentage of previously unidentified moderate (43.3%) to severe (36.7%) sleep apnea in patients presenting with COPD exacerbation.

The mean age of our study population was 72.6 ± 14.3 SD. As such, studies have shown that the coexistence of the AHI may be higher in people over 70 years of age and may reach up to 30% [31]. Previous research discusses that in patients with COPD, OSA, and hypercapnia, NIV can be started with a positive result [32].

Furthermore, OSA is frequently associated with hypertension, atrial fibrillation, stroke, heart failure, and sudden death. We found that 33% of our patients had arterial hypertension and 13.3% diabetes mellitus-type II [33]. Our study also included a patient with hypercapnic acute respiratory failure with moderate to severe obstruction with forced expiratory volume 1.0 sec (FEV1) of less than one liter.

Based on the 2007 recommendations of the Portable Monitoring Task Force of the American Academy of Sleep Medicine [11], we used portable monitors as an alternative to PSG for the diagnosis of AHI, because our patients had severe clinical conditions and were incapable of moving to a sleep laboratory facility [34, 35]. In addition, we found that patients with severe AHI had longer hospitalization stays and more days requiring NIV. Moreover, two patients with AHI and OI classified as moderate to severe required intubation and IMV.

On the other hand, all of our patients were ventilated with BiPAP S/T-AVAPS for the management of acute hypercapnic respiratory failure, which could influence the outcome due to the ability of AVAPS to open the upper airway and eliminate obstructive apnea [36] Additionally, we analyzed the results of respiratory polygraphy and spirometry at the time of hospital discharge of patients who were not previously evaluated with sleep studies.

### 4.1. Limitations

1. Patients who did not respond to NIV and were switched to conventional IMV through orotracheal intubation and patients who died were excluded from the study.
2. We used portable monitoring devices, which could affect the diagnostic precision of patients with special comorbidities.
3. The ApneaLink device cannot be used for studying OSA since it is a single-channel screening tool for sleep apnea that measures airflow through a nasal cannula connected to a pressure transducer, providing an Apnea-Hypopnea Index (AHI) based on recording time. The device cannot distinguish obstructive apneas from central or mixed ones. However, the employed portable monitor complies with the Standards of the Practice Committee of the American Sleep Disorders Association that establishes that portable monitoring is an adequate method for clinical therapeutic decision making, with an agreement with polysomnography (PSG) of ∼90% [37, 38].

In conclusion, we found a high presence of sleep apnea in patients with acute hypercapnic respiratory failure and COPD exacerbations that require NIV; this association could influence successful results when using AVAPS mode as a ventilatory strategy. However, larger studies should be performed to address the outcome implications of higher AHI scores in this patient population.

## Figures and Tables

**Table 1:** Baseline characteristics of the study population.

Characteristics	Means ± SD
Age (years)	72.6 ± 14.3
Sex (F/M)	13 (43.3%)
	17 (56.7%)
Weight (Kg)	63.04 ± 14.28
Height (cm)	157.5 ± 10.3
BMI	25.4 ± 5.2
Neck circumference (cm)	48.8 ± 5.4
Mallampati score	1.6 ± 0.6
Epworth sleepiness scale	9.6 ± 5.90
Snoring	No, 19 (63.3%)
	Yes, 11 (36.7%)
Apnea reported by patient	No, 25 (83.3%)
	Yes, 5 (16.7%)
Days of stay in ICU	8.6 ± 3.11
Days of NIV	5.6 ± 2.76
SBP (systolic blood pressure) (mmHg)	130.8 ± 17.1
DBP (diastolic blood pressure (mmHg)	75.2 ± 9.4
HR (heart rate)	89.1 ± 12
RR (respiratory rate)	25.7 ± 4.7
SaO_2_ oximeter (%)	94 ± 3
AHI (Apnea-Hypopnea Index)	14.3 ± 14
IDO (Oxygenation Index)	6.6 ± 5.9
SO_2_ average (%)	91.9 ± 4.3
SO_2_ lower (%)	73.4 ± 15.2
pH	7.32 ± 0.02
pCO_2_ (mmHg)	53.7 ± 10.4
PO_2_ (mmHg)	75.4 ± 13.8
HCO_3_ (mmol/L)	27.2 ± 6
EB	2.9 ± 5.3
PaO_2_/FiO_2_ (mmHg)	197.3 ± 38.9
FVC (pre)	1.4 ± 0.53
FEV^∗^1,0 (pre)	0.88 ± 0.4
FEV^∗^1,0/FVC (pre)	49.8 ± 26.6
PEF (pre)	2.1 ± 1.02
FVC (post)	1.45 ± 0.51
FEV^∗^1,0 (post)	0.9 ± 0.37
FEV^∗^1,0/FVC (pot)	50.8 ± 24.4
PEF (post)	2.4 ± 1.4
Tidal volume (ml)	447.7 ± 67.6
Levels of EPAP (cmH_2_O)	6.1 ± 0.4
Levels of IPAP programmed maximum (cmH_2_O)	19.7 ± 0.9
Levels of IPAP (cmH_2_O)	13.1 ± 4.9
Leak (cmH_2_O)	17.9 ± 7.2
Inspiratory time (sec)	0.8 ± 0.2
*V*_min_ (L/min)	10.3 ± 4.1
RAMP (msec)	3 ± 0.2
Comorbidity	No, 16 (53.3%)
	Yes, 14 (46.7%)
Diabetes mellitus-type II	4 (13.3%)
Hypertension	10 (33.3%)
None	16 (53.3%%)
Orotracheal intubation	Yes, 2 (6.7%)
	Not 28 (93.3%)
Affected lung quadrants	None (13) (43.3%)
	1 quadrant (13) (43.3%)
	2 quadrants (4) (13.3%)
AHI (Apnea-Hypopnea Index)	<5 (6) (20%)
	5–15 (13) (43.3%)
	>15 (11) (36.7%)

Continuous variables are presented as mean ± SD. Categorical variables are presented as No. (%).

**Table 2:** The characteristics of group's severity according to AHI.

Variables	Apnea-Hypopnea Index <5	Apnea-Hypopnea Index 5–15	Apnea-Hypopnea Index >15	*p* Value
	(*n* = 6)	(*n* = 13)	(*n* = 11)	
Age (years)	66.5 ± 18.7	71.7 ± 14.5	77 ± 11.4	0.354
Weight (Kg)	64 ± 6.3	58.4 ± 10.2	67.9 ± 19.8	0.275
Height (cm)	157.3 ± 8.8	156.6 ± 9.9	158.7 ± 12.2	0.891
BMI	26.2 ± 5.5	23.7 ± 2.8	26.9 ± 6.9	0.291
Days of stay in ICU	7.8 ± 3.2	9.08 ± 3.9	8.4 ± 2.0	0.722
Days of NIV	4.8 ± 1,7	5.9 ± 3.8	5.8 ± 1.7	0.721
SBP (systolic blood pressure) (mmHg)	133.3 ± 11.2	128.8 ± 14.6	131.8 ± 22.9	0.853
DBP **(**diastolic blood pressure (mmHg)	78.1 ± 6.1	74 ± 6.5	75 ± 13.6	0.685
HR (heart rate)	99 ± 18.6	86.6 ± 5.9	86.6 ± 11.6	0.076
RR (respiratory rate)	25.8 ± 2.9	26.8 ± 4.8	24.2 ± 5.4	0.432
SaO_2_	94.6 ± 3.1	92.6 ± 1.7	94.5 ± 3.1	0.155
Neck circumference (cm)	42.4 ± 2,9	49.1 ± 5.2	52 ± 3.4	0.001^∗^
Epworth scale	2 ± 1	9.4 ± 4,8	14.2 ± 3.6	0.001^∗^
Mallampati score	1.5 ± 0,5	1.4 ± 0,5	1.9 ± 0,8	0.001^∗^
Snoring	3 of 6	4 of 13	4 of 11	0.700
Apnea reported by patient	0 of 6	2 of 13	8 of 11	0.300
Comorbidity	3 of 6	6 of 13	5 of 11	0.900
pH	7.33 ± 0.04	7.33 ± 0.02	7.33 ± 0,03	0.913
pCO_2_ (mmHg)	47.8 ± 11.5	55.53 ± 12.1	54.7 ± 6.7	0.307
PO_2_ (mmHg)	77.4 ± 11.1	72.3 ± 13.4	77.8 ± 16.0	0.588
HCO_3_ (mmol/L)	24.3 ± 4.9	27.7 ± 6.8	28.1 ± 5.7	0.433
EB	0.27 ± 4,5	3.64 ± 6,1	3.3 ± 4,6	0.419
PaO_2_/FiO_2_ (mmHg)	202.6 ± 21,6	185 ± 40.7	208.8 ± 42.6	0.318
FVC (pre)	1.32 ± 0,3	1.33 ± 0,5	1.54 ± 0,7	0.595
FEV^∗^1,0 (pre)	0.8 ± 0,2	0.82 ± 0,4	0.99 ± 0,5	0.520
FEV^∗^1,0/FVC (pre)	58.7 ± 10.2	61.2 ± 15.0	61.2 ± 12,2	0.921
PEF (pre)	1.74 ± 0,7	2.02 ± 0,8	2.42 ± 1,3	0.406
FVC (post)	1.36 ± 0,3	1.37 ± 0,5	1.59 ± 0,6	0.525
FEV^∗^1,0 (post)	0.82 ± 0,3	0.83 ± 0,4	1 ± 0.5	0.517
FEV^∗^1,0/FVC (post)	0.68 ± 7,2	1.98 ± 7,4	2.13 ± 5,1	0.901
PEF (post)	1,94 ± 0,8	2,6 ± 2,0	2,48 ± 1,1	0.677
Tidal volume (ml)	546.6 ± 231.7	413,8 ± 81.7	513.6 ± 238.8	0.262
Levels of EPAP (cmH2O)	6 ± 0	6 ± 0	6.1 ± 0.6	0.436
Levels of maximum programmed IPAP (cmH_2_O)	20 ± 1.6	19.5 ± 0.9	19.8 ± 0,6	0.668
Levels of IPAP patient (cmH_2_O)	16.3 ± 2.7	16.6 ± 2.2	16.8 ± 1.3	0.875
Leak (cmH_2_O)	20.1 ± 6.2	17.6 ± 7.1	16.9 ± 8.1	0.678
Inspiratory time (sec)	0.78 ± 0.2	0.88 ± 0.2	0.79 ± 0.1	0.335
*V*_min_ (L/min)	10.6 ± 1.4	8.6 ± 3.0	12 ± 5.6	0.129
RAMP (msec)	3 ± 0	3 ± 0.3	3 ± 0	0.536

^∗^Statistical significance at *p* < 0.05: statistically significant differences in neck circumference (cm), Epworth scale, and Mallampati score.

**Table 3:** Intubation events according to Apnea-Hypopnea and Deoxygenation Indices.

Events	Apnea-Hypopnea Index			IDO Index		
	<5	5 a 15	>15	<5	5 a 15	>15
Intubation	—	1 (50%)	1 (50%)	—	2(100%)	—
No intubation	6 (21.4%)	12 (42.9%)	10 (35.7%)	14 (50%)	12 (42.9%)	2 (7.1%)
Days of stay in ICU	7,8 ± 3.1	9 ± 3.9	8.4 ± 1,9	8.4 ± 3,7	9 ± 2.6	7 ± 1.4
Days of NIV	4.8 ± 1.7	5.9 ± 3.8	5.8 ± 1.6	5.3 ± 3.2	6 ± 2.4	5 ± 1.4

## Data Availability

The Excel table data used to support the findings of this study are available from the corresponding authors upon request, killenbrio@hotmail.com and ucihospitalbabahoyo@gmail.com.

## Abbreviations

ABG: Arterial blood gas
AHI: Apnea-Hypopnea Index
AHS: Apnea-hypopnea syndrome
AHRF: Acute hypercapnic respiratory failure
ARF: Acute respiratory failure
AVAPS: Average volume-assured pressure support
BiPAP S/T-AVAPS: Bilevel positive airway and average volume-assured pressure support
COPD: Chronic obstructive pulmonary disease
DBP: Diastolic blood pressure
DNR: Do-not-resuscitate
EB: Excess base
EEG: Electroencephalogram
EVT: Exhaled tidal volume
EPA: Expiratory positive airway pressure
FEV^∗^1, 0: Forced expiratory volume 1, 0 sec
FEV^∗^1, 0/FVC: The ratio of FEV^∗^1.0 and the forced vital capacity
FVC: Forced vital capacity
HCO_3_ type: Sets the concentration of buffer or blood bicarbonate
HR: Heart rate
ICU: Intensive care unit
IPAP: Inspiratory positive airway pressure
NIV: Noninvasive mechanical ventilation
OSA: Obstructive sleep apnea
pCO_2_: Carbon dioxide concentration
pH: The negative algorithm of the hydrogen ion concentration
PO_2_: Partial pressure of arterial oxygen
PSG: Polysomnography
RR: Respiratory rate
SaO_2_: Arterial oxygen saturation
SBP: Systolic blood pressure
SD: Standard deviation
TV: Tidal volume
*V*_min_: Minute volume.
